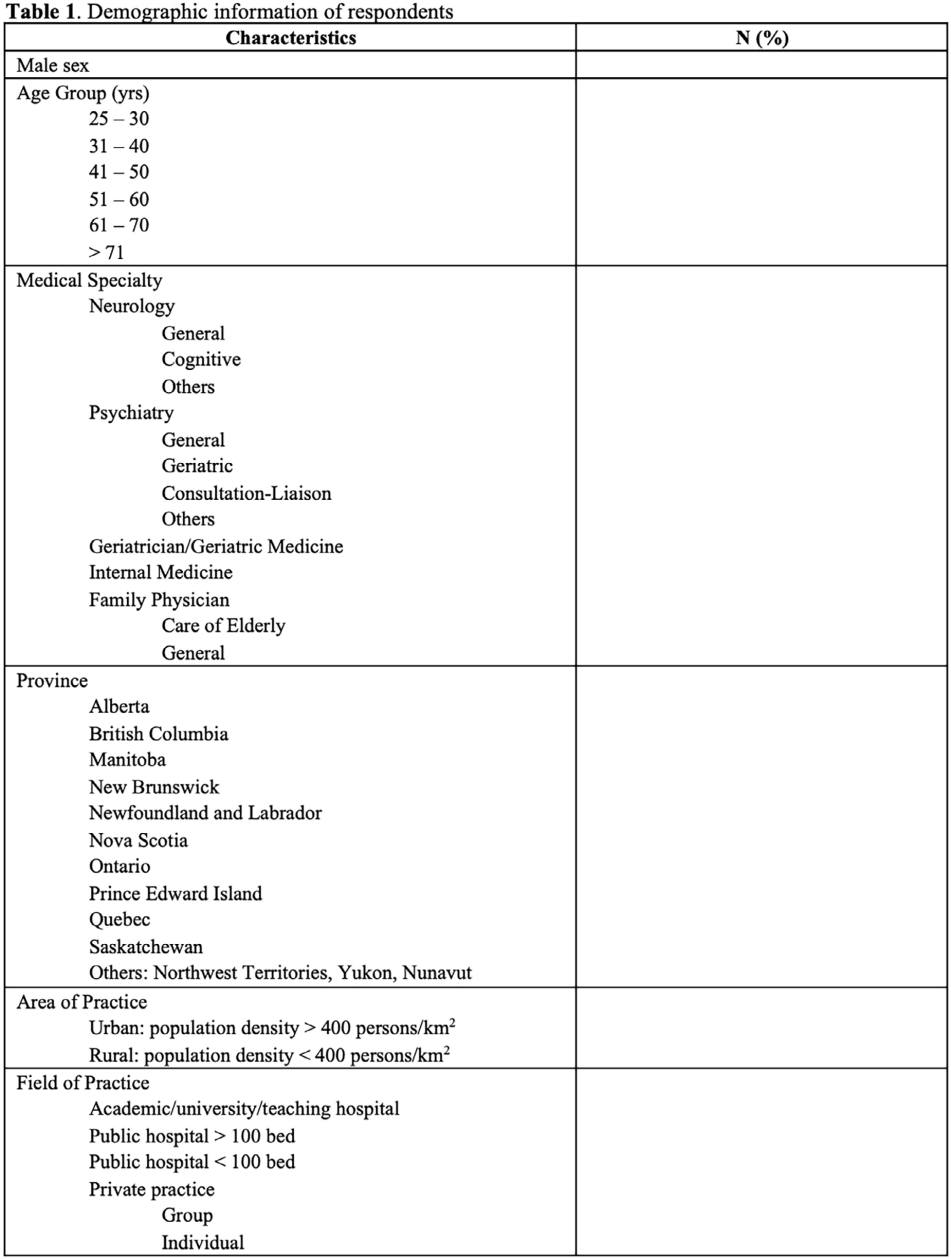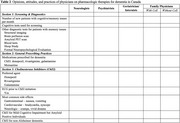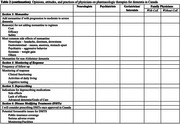# The Opinions, Attitudes, and Practices of Physicians Regarding Pharmacologic Therapies for Dementia in Canada

**DOI:** 10.1002/alz70860_103577

**Published:** 2025-12-23

**Authors:** Ferron F Ocampo, Joey Champigny, Krista L Lanctôt, Mario Masellis, Sara Mitchell

**Affiliations:** ^1^ Department of Psychiatry, University of Toronto, Toronto, ON, Canada; ^2^ MINT Memory Clinics, The Centre for Family Medicine Family Health Team, Kitchener, ON, Canada; ^3^ Sunnybrook Health Sciences Centre, Toronto, ON, Canada; ^4^ Hurvitz Brain Sciences Program, Sunnybrook Research Institute, Toronto, ON, Canada; ^5^ Department of Pharmacology and Toxicology, University of Toronto, Toronto, ON, Canada; ^6^ Division of Neurology, Department of Medicine, Sunnybrook Health Sciences Centre, Toronto, ON, Canada; ^7^ Division of Neurology, Department of Medicine, University of Toronto, Toronto, ON, Canada

## Abstract

**Background:**

Despite the approval of disease‐modifying therapies (DMTs) in select countries worldwide, cholinesterase inhibitors (ChEIs) and the NMDA receptor antagonist memantine remain the only approved pharmacologic agents for symptomatic treatment of dementia in Canada. The absence of standardized prescribing guidelines for these medications has led to significant variability in their use among physicians who care for persons living with dementia (PLWD). Furthermore, perspectives among specialists also differ regarding the significance of DMT outcomes relative to their potential risks. Our study aims to explore the opinions, attitudes, and prescribing practices of Canadian physicians concerning ChEIs, memantine, and integration of emerging DMTs in Canada.

**Method:**

In this cross‐sectional study, we developed a semi‐structured questionnaire based on published recommendation‐based consensus as well as a quality‐weighted literature review on pharmacologic therapies for dementia. The questionnaire is comprised of the following sections: *demographic information, screening tools and diagnostics for dementia, general prescribing practices, prescribing ChEIs and memantine, monitoring of response, deprescribing*, and *disease‐modifying therapies*. The questionnaire will be sent electronically to physicians involved in the care of PLWD, including general and cognitive neurologists, geriatric psychiatrists, geriatricians, internists, and family physicians with/without care of the elderly (CoE) training across Canada. Continuous, and categorical variables will be analyzed using means and proportions. Inferential statistics will be used to explore the differences in responses across the different medical specialties.

**Result:**

We will send the questionnaires to at least 20 physicians per specialty across the different provinces of Canada and we will aim for a response rate of 30% (target number of participants=50), with at least 1 representative from each specialty. The demographic information of respondents will be presented as shown in Table 1. The responses for each section of the questionnaire will be presented as shown in Table 2. We will present the final results during the AAIC 2025 in Toronto, Canada.

**Conclusion:**

The results of this study will be utilized to create the initial (round one) questionnaire for a Delphi study to establish consensus guidelines regarding the use of ChEIs and memantine for the treatment of PLWD in Canada, as well as to inform disease‐modifying medication rollout in Canada once appropriate.